# The Fluorescent Detection of Alkaline Phosphatase Based on Iron Nanoclusters and a Manganese Dioxide Nanosheet

**DOI:** 10.3390/s25020585

**Published:** 2025-01-20

**Authors:** Liang Zhao, Xinyue Liu, Xinwen Zhang, Siyu Liu, Jiazhen Wu

**Affiliations:** 1 College of Life and Health Sciences, Northeastern University, Shenyang 110169, China; m15703450938@163.com (L.Z.); liuxinyue0301@163.com (X.L.); 2 College of Medicine and Biological Information Engineering, Northeastern University, Shenyang 110057, China; mkbkxy@163.com; 3 Department of Obstetrics and Gynecology, Shengjing Hospital of China Medical University, Shenyang 110004, China

**Keywords:** fluorescence, detection, ALP, iron nanocluster, MnO_2_ NS

## Abstract

Fluorescent iron nanoclusters are emerging fluorescent nanomaterials. Herein, we synthesized hemoglobin-coated iron nanoclusters (Hb−Fe NCs) with a significant fluorescence emission peak at 615 nm and investigated the inner-filter effect of fluorescence induced by a manganese dioxide nanosheet (MnO_2_ NS). The fluorescence quenching of Hb−Fe NCs by a MnO_2_ NS can be significantly reversed by the addition of ascorbic acid. On the basis of fluorescent recovery by ascorbic acid, we proposed a system that consisted of Hb−Fe NCs, a MnO_2_ NS and ascorbate phosphate, and the proposed system was successfully used for alkaline phosphatase (ALP) detection in the range of 0–20 μg/mL based on the significant fluorescence recovery achieved.

## 1. Introduction

Metal nanoclusters are typically composed of several to dozens of metal atoms [1]. Due to their small size, which is comparable to the Fermi wavelength of electrons, metal nanoclusters have discrete energy levels similar to molecules, allowing them to undergo electronic transitions and emit light under external light excitation [2]. Generally, metal nanoclusters have the advantages of size-dependent luminescence, a long fluorescence lifetime, a large Stokes shift, a simple preparation method and good biocompatibility and have attracted increasing attention in the fields of biomedicine, optical materials and catalytic materials [3]. For instance, Xu et al. developed a sensor array composed of various copper nanoclusters, which can detect twelve kinds of metal ions, humic substances, lipids, amino acids and lignans [4]. Xi et al. developed a three-dimensional mixed ion detection system based on bovine serum albumin-encapsulated gold nanoclusters and various dopamine levels as non-specific receptors [5]. However, most studies on metal nanoclusters focused on noble metal nanoclusters consisting of gold, silver, copper or platinum atoms [6,7]. Limited resources and high costs restricted their applications. In contrast, iron resources are low-cost and are widely available worldwide [8]. Therefore, zero-valent iron nanomaterials are becoming an ideal metal for creating nanoclusters [9]. Due to the high reactive activity of a zero-valent iron atom, zero-valent iron nanomaterials have typically been used to treat contaminants in various environments and have also been used as an antibacterial material [10,11]. However, very few studies have applied iron nanoclusters for the purpose of fluorescence detection [12,13].

A manganese dioxide nanosheet (MnO_2_ NS) is a layered transition-metal dioxide nanomaterial, and the Mn atom in the MnO_2_ NS coordinates octahedrally with the nearest six oxygen atoms [14]. Similarly to graphene, the MnO_2_ NS is a typical two-dimensional nanomaterial, which is widely used in biosensors, bioimaging and drug delivery due to its large surface area, wide absorption band, unique catalytic properties, etc. [15,16].

Alkaline phosphatase (ALP), a vital hydrolytic enzyme, is prevalent in bodily fluids and tissue organs such as the liver, intestines, kidneys, etc., in various mammals [17]. ALP mainly participates in catalyzing the hydrolysis of phosphate monoester groups [18]. In addition, ALP is recognized as a biomarker for many diseases such as rickets, malignant bone tumors, breast cancer, jaundice, diabetes, etc. [19]. An abnormal level of ALP activity in humans usually reveals potential health risks. Therefore, it is necessary to establish fast, accurate, sensitive and widely applicable detection methods to detect the ALP level [20]. Recently, various fluorescent probes have been prepared and used for ALP detection [21,22,23]. For instance, Feng et al. constructed a calcein–Ce^3+^–phosphopeptide system, which was used for the fluorescent detection of ALP based on the fact that ALP could catalyze the phosphopeptide to generate phosphate [21]. The quenched fluorescence of calcein can be recovered due to the higher affinity between phosphate and Ce^3+^. Mao et al. prepared cyan-emitting fluorescent Si dots and demonstrated the synergistic quenching effect induced by 4−nitrophenyl phosphate [22]. The Si dots–4–nitrophenyl phosphate system was utilized for the turn-off detection of ALP on the basis of the ALP-catalyzed hydrolysis of 4-nitrophenyl phosphate.

**Scheme 1:**
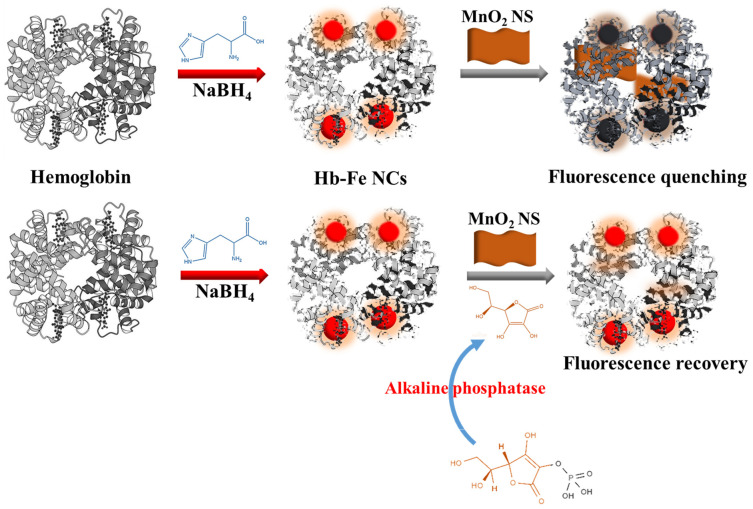
The illustration of fluorescent detection of alkaline phosphatase utilizing Hb−Fe NCs and MnO_2_ NS.

In this work, we prepared the red-emitting fluorescence probe Hb−Fe NCs in a solution. We began by describing the Hb−Fe NCs and MnO_2_ NS system and then demonstrated the inner-filter effect (IFE) of fluorescence between Hb−Fe NCs and the MnO_2_ NS [24,25,26]. Furthermore, we investigated the fluorescence recovery of Hb−Fe NCs and the MnO_2_ NS system induced by ascorbic acid (VC) based on the VC-mediated degradation of MnO_2_ NS. In view of the fact that ALP can catalyze sodium ascorbate phosphate (AP) to generate VC, as shown in Scheme 1, we proposed a system composed of Hb−Fe NCs, MnO_2_ NS and AP (Hb−Fe NCs−MnO_2_ NS−AP) for the fluorescent detection of ALP on the basis of the transformation from AP to VC induced by ALP.

## 2. Experiments

### 2.1. Chemicals and Reagents

Bovine hemoglobin was procured from Aladdin Reagent, located in Shanghai, China. Pepsin and trypsin were sourced from Shanghai Yubo (Gen−view) Biotechnology Company (Shanghai, China). Fetal bovine serum, ascorbic acid (VC), sodium ascorbate phosphate (AP), alkaline phosphatase, phenylalanine (Phe), tryptophan (Trp), threonine (Thr), citric acid (CA), proline (Pro), glucose (Glu), and L−histidine were obtained from Dingguo Biotechnology Company (Beijing, China). Anhydrous ethanol, sodium borohydride (NaBH_4_), Tris−HCl, sodium hydroxide (NaOH), sodium chloride (NaCl), magnesium chloride (MgCl_2_), calcium chloride (CaCl_2_) were acquired from Shanghai Hutest Laboratory Equipment Co., Ltd. (Shanghai, China). All reagents utilized in this study were at least of analytical grade, and did not necessitate further purification. The resistivity of the deionized water employed in the experiments exceeded 18 Ω·m.

### 2.2. Synthesis of Hb−Fe NCs

The synthesis process was performed based on a previous report with modifications [12]. Hemoglobin powder (40 mg) was ultrasonically dispersed in 4 mL of deionized water. Subsequently, 5.0 mL of L−histidine solution (0.15 mol·L^−1^) and 15.0 mL of ethanol solution containing NaBH_4_ (0.021 mol·L^−1^) were added to the aforementioned solution, which was then stirred for six hours at room temperature. Upon completion of the reaction, a beige solution was obtained. This solution was further centrifuged at 10,000 rpm for 10 min, and the supernatant was retained. The supernatant underwent purification via ultrafiltration centrifugation at 7000 rpm (3000 KD, Millipore, Boston, MA, USA) to yield a purified Hb−Fe NC solution. Ultimately, Hb−Fe NCs powder was acquired through vacuum freeze-drying.

### 2.3. Synthesis of MnO_2_ NS

The synthesis of MnO_2_ NS was conducted according to a previous report with some modifications [24]. MnCl_2_·4H_2_O (0.5935 g) was dissolved in 10 mL of deionized water, followed by the addition of 20 mL of an aqueous solution containing tetramethylammonium hydroxide (0.6 mol/L) and hydrogen peroxide (3.0 wt%). The resulting mixture was continuously stirred at room temperature for 12 hours, during which a significant amount of bubbles was produced. Subsequently, the dark brown solution obtained was centrifuged at 12,000 rpm for 10 min to collect the precipitate, which was then washed with ethanol and water three times. Finally, MnO_2_ NS powder was obtained through vacuum freeze-drying.

### 2.4. The Effect of Ascorbic Acid on Hb−Fe NCs and MnO_2_ NS System

In a centrifuge tube, 40 μL of Tris−HCl solution (pH 7.4, 100 mM), 40 μL of Hb−Fe NC solution (8.25 mg·mL^−1^), 40 μL of MnO_2_ NS solution (0.25 mg·mL^−1^), and varying amounts of VC solution were sequentially added, followed by dilution to 200 μL with deionized water. After incubating the mixture at room temperature for 60 min, the fluorescence emission spectra and UV−Vis absorption spectra of the mixture were measured using a Biotek Synergy^TM^ H1 microplate reader (BioTek, Winooski, VT, USA).

### 2.5. Detection of ALP Based on Hb−Fe NCs and MnO_2_ NS System

In this study, a series of solutions were prepared for the detection of ALP. Specifically, 40 μL of Tris−HCl solution (pH 7.4, 100 mM), 40 μL of Hb−Fe NC solution (8.25 mg·mL^−1^), 40 μL of MnO_2_ NS solution (0.25 mg·mL^−1^), and 10 μL of AP solution (10 mM) were sequentially added to a centrifuge tube. Various volumes of ALP solution were then incorporated, and the total volume was adjusted to 200 μL with deionized water. Following a 60-min incubation period at ambient temperature, the fluorescence emission and UV−Vis absorption spectra of the resultant mixture were analyzed using a Biotek Synergy^TM^ H1 microplate reader (BioTek, Winooski, VT, USA).

## 3. Results and Discussions

### 3.1. The Synthesis of Hb−Fe NCs and MnO_2_ NS

**Figure 1:**
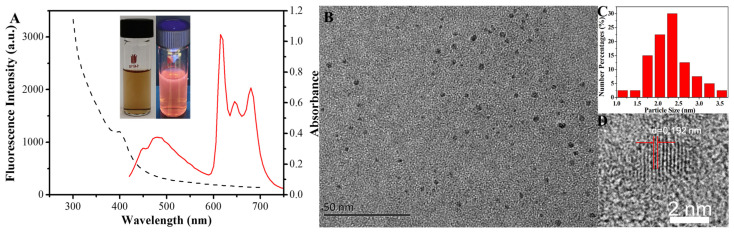
(**A**) The fluorescent emission spectra of Hb−Fe NCs (solid line) and UV−Vis absorption spectra of Hb−Fe NCs (dash line). The inset presents an image of the Hb−Fe NC solution illuminated by visible light (left) and UV light (right). (**B**) The TEM images of Hb−Fe NCs. (**C**) The statistical data regarding the particle distribution of Hb−Fe NCs. (**D**) The high-resolution TEM image of Hb−Fe NCs.

**Figure 2:**
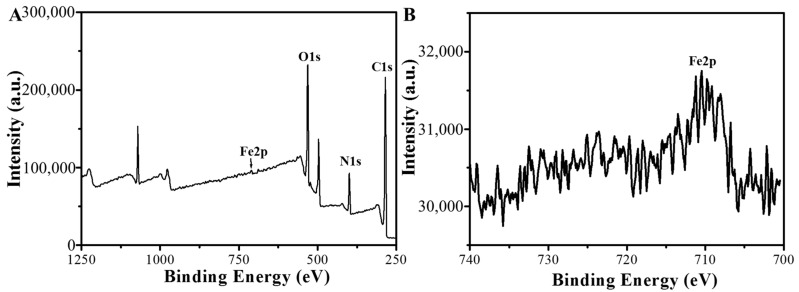
(**A**) Total XPS spectrum of Hb−Fe NCs. (**B**) High-resolution Fe2p XPS spectrum.

**Figure 3:**
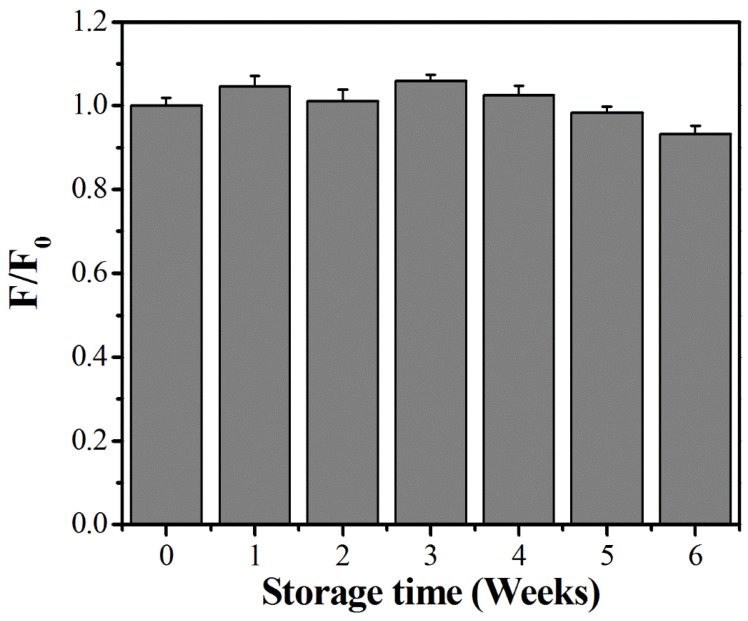
The fluorescence intensity at 615 nm of the Hb−Fe NC solution was monitored over a six-week period while stored at room temperature. F_0_ presents the initial fluorescence intensity at 615 nm of Hb−Fe NC solution, and F denotes the fluorescence intensity at 615 nm of Hb−Fe NC solution measured at various time points throughout the six weeks.

As illustrated in Scheme 1, Hb−Fe NCs were synthesized utilizing hemoglobin as the iron source, L−histidine as the extractant, and sodium borohydride as the reducing agent. Figure 1A demonstrates that the synthesized Hb−Fe NCs exhibited multiple fluorescence emission peaks at 480 nm, 615 nm, 650 nm, and 680 nm upon excitation at 400 nm. The Hb−Fe NC solution displayed a vivid red coloration under ultraviolet illumination (Figure 1A Inset). Additionally, the UV−Vis absorption spectra presented in Figure 1A revealed a prominent absorption peak at 395 nm. The quantum yield of the Hb−Fe NCs was determined to be 8.2%, using rhodamine 6G as a reference standard. Transmission electron microscopy (TEM) was employed to assess the morphology of the Hb−Fe NCs. As shown in Figure 1C, the Hb−Fe NCs appeared as spherical particles with a size distribution ranging from 1.22 to 3.55 nm, with a mean size of 2.29 nm. High-resolution TEM imaging indicated that the Hb−Fe NCs exhibited high crystallinity, characterized by a lattice distance of 0.192 nm (Figure 1D). X-ray photoelectron spectroscopy (XPS) analysis confirmed the presence of carbon (C), nitrogen (N), oxygen (O), and iron (Fe) elements in the Hb−Fe NCs (Figure 2A). The high-resolution XPS spectrum for the Fe2p region indicated a signal around 709 eV, which is attributed to zero-valent iron (Figure 2B). These findings collectively affirm the successful synthesis of fluorescent iron nanoclusters, Hb−Fe NCs. To evaluate the stability of the Hb−Fe NCs, fluorescence measurements at 615 nm were conducted over a six-week period. As depicted in Figure 3, the fluorescence intensity of the Hb−Fe NC solution remained relatively stable for four weeks when stored at room temperature, with a slight decrease observed during the fifth and sixth weeks. Consequently, the synthesized Hb−Fe NCs demonstrate potential as a fluorescent probe for future applications.

**Figure 4:**
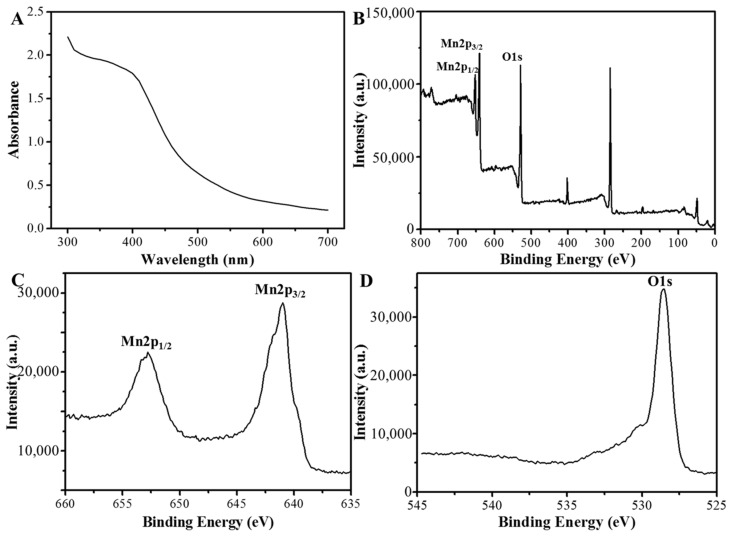
(**A**) The UV−Vis absorption spectra of MnO_2_ NS. (**B**) Total XPS spectrum of MnO_2_ NS. (**C**) High-resolution Mn2p XPS spectrum. (**D**) High-resolution O1s XPS spectrum.

**Figure 5:**
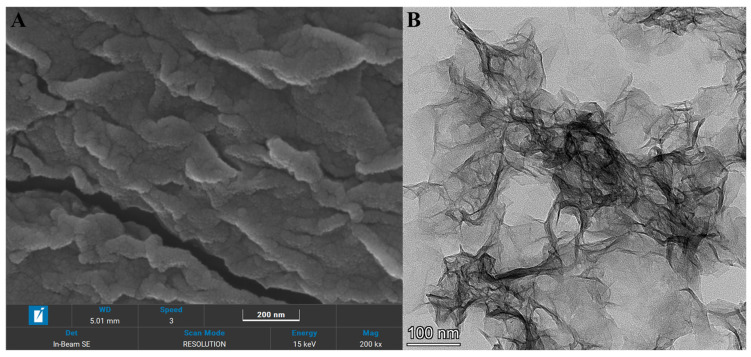
(**A**) SEM images of MnO_2_ NS. (**B**) TEM images of MnO_2_ NS.

The MnO_2_ NS were synthesized following established methodologies with certain modifications [27]. The absorption spectrum of the MnO_2_ NS exhibited a characteristic peak at approximately 380 nm, which is indicative of the MnO_2_ NS (Figure 4A) [28]. To further validate the composition and oxidation state, X-ray photoelectron spectroscopy (XPS) was employed. The results presented in Figure 4B confirmed the presence of manganese (Mn) (Mn2p) and oxygen (O) (O1s) elements. The high-resolution XPS spectrum for Mn2p revealed two distinct binding energies at 652.8 eV and 641.2 eV, corresponding to Mn2p_1/2_ and Mn2p_3/2_, respectively. The observed energy separation of 11.6 eV suggests that manganese exists in a tetravalent state within the MnO_2_ NS (Figure 4C) [28]. Additionally, scanning electron microscopy (SEM) and transmission electron microscopy (TEM) images of the MnO_2_ NS are presented in Figure 5, illustrating a plate-like aggregation in the SEM image and a thin-layered structure in the TEM image. Collectively, these findings substantiate the successful synthesis of MnO_2_ NS.

**Figure 6:**
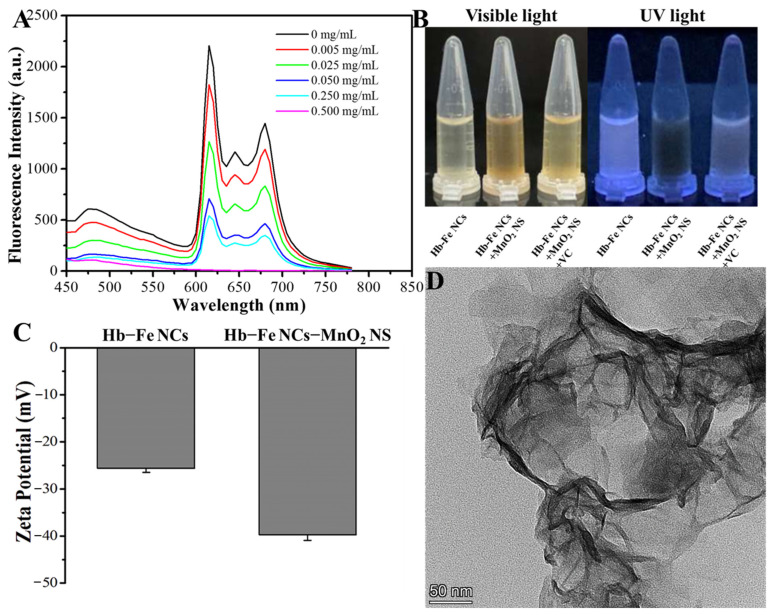
(**A**) The fluorescence emission spectra of Hb−Fe NCs incubated with different MnO_2_ NS concentrations for 30 min. (**B**) Hb−Fe NC solution, Hb−Fe NCs−MnO_2_ NS, and Hb−Fe NCs mixed with 0.050 mg/mL of MnO_2_ NS and 0.5 mM of VC solution under both visible light and UV light. (**C**) Zeta potential measurements for the Hb−Fe NC solution and the Hb−Fe NCs−MnO_2_ NS. (**D**) TEM image of Hb−Fe NCs−MnO_2_ NS.

As illustrated in Scheme 1, we conducted a detailed examination of the fluorescence alterations in Hb−Fe NCs when incubated with MnO_2_ NS. Figure 6A illustrates a gradual reduction in fluorescence emission peaks at 480 nm and 615 nm as the concentration of MnO_2_ NS increased from 0 to 0.500 mg/mL, following excitation at 400 nm (with concentrations of 0, 0.005, 0.025, 0.050, 0.250, and 0.500 mg/mL, respectively). Figure 6B presents images of the Hb−Fe NC solution, the Hb−Fe NCs mixed with 0.050 mg/mL of MnO_2_ NS solution (designated as Hb−Fe NCs−MnO_2_ NS), and the Hb−Fe NCs combined with 0.050 mg/mL of MnO_2_ NS and 0.5 mM of VC solution, observed under both visible and ultraviolet (UV) light. The original Hb−Fe NC solution appeared colorless, while the Hb−Fe NCs−MnO_2_ NS solution exhibited a light brown coloration. Under UV light, the original Hb−Fe NC solution displayed red fluorescence, which was significantly quenched in the presence of MnO_2_ NS, observed after the mixture with 0.050 mg/mL of MnO_2_ NS under UV light. The zeta potential of both the Hb−Fe NCs and the Hb−Fe NCs−MnO_2_ NS solution was assessed. As illustrated in Figure 6C, the zeta potential exhibited a shift from −25.6 mV to −39.7 mV following the incorporation of MnO_2_ NS. Additionally, Figure 6D presents a TEM image of the Hb−Fe NCs−MnO_2_ NS, revealing that the tiny spherical nanoparticles (Hb−Fe NCs) predominantly adhered to the surface of the layered MnO_2_ NS.

**Figure 7:**
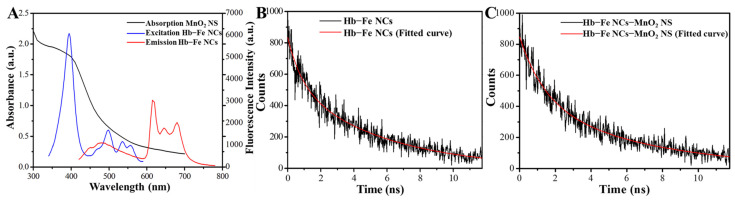
(**A**) The UV−Vis absorption spectra of MnO_2_ NS are represented by the black line, the fluorescence excitation spectra with 615 nm emission are represented by the blue line, and fluorescence emission spectra with 400 nm excitation are represented by the red line. The fluorescence decay curve and fitted curve of Hb−Fe NC solutions (**B**) and Hb−Fe NC solutions incubated with 0.050 mg/mL of MnO_2_ NS (**C**). Excitation wavelength was 400 nm, and emission wavelength was 615 nm.

Previous studies have indicated that MnO_2_ NSs serve as effective quenchers for fluorescent probes, attributed to their broad absorption spectrum [13,24,25,26,27]. The primary mechanisms of quenching associated with MnO_2_ NS include fluorescence resonance energy transfer (FRET) and inner-filter effect (IFE). Figure 7A illustrates the fluorescence excitation and emission spectra of the Hb−Fe NCs, alongside the UV−Vis spectrum of MnO_2_ NS. Notably, the fluorescence excitation spectrum of the Hb−NCs exhibits a pronounced peak at 395 nm. The absorption spectrum of MnO_2_ NS significantly overlaps with the excitation peak of Hb−Fe NCs at 395 nm, while there is a minor overlap with the emission peak at 615 nm. Additionally, the fluorescence lifetime of Hb−Fe NCs was assessed both in the absence and presence of MnO_2_ NS at a concentration of 0.050 mg/mL. The results, depicted in Figure 7B,C, demonstrate that the fluorescence decay curves can be accurately modeled using the following equation:

I_(t)_ = A_1_exp(−t/τ_1_) + A_2_exp(−t/τ_2_) (1)

The pertinent parameters are presented in Table 1. The fluorescence lifetime (τ) of the Hb−Fe NCs, both in the absence and presence of MnO_2_ nanosheet, was determined to be 5.20 ns and 5.71 ns, respectively, as calculated using the following equation:

τ = (A_1_τ_1_^2^ + A_2_τ_2_^2^)/(A_1_τ_1_ + A_2_τ_2_) (2)

Considering the minimal variation in the fluorescence lifetime of Hb−Fe NCs following the introduction of MnO_2_ NS, along with the negligible overlap between the fluorescence emission peak at 615 nm of Hb-NCs and the absorption band of MnO_2_ NS, it can be concluded that FRET is not a contributing factor to the observed quenching mechanism [13,29].

In contrast, IFE represents a non-irradiative energy conversion model within fluorescence techniques and has demonstrated various applications in chemical and biosensing domains [29]. The pronounced decrease in fluorescence intensity of Hb−Fe NCs at 615 nm upon the addition of MnO_2_ NS can be ascribed to the IFE mechanism, which is supported by the substantial overlap between the fluorescence excitation peak at 395 nm of Hb−Fe NCs and the absorption band of MnO_2_ NS [29,30].

**Figure 8:**
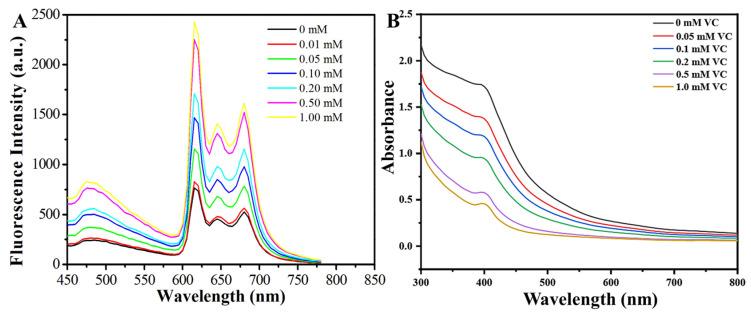
(**A**) The fluorescence emission spectra of Hb−Fe NCs incubated with 0.050 mg/mL of MnO_2_ NS and varying concentrations of VC for 30 min. (**B**) The UV−Vis absorption spectra of Hb−Fe NCs incubated with 0.050 mg/mL of MnO_2_ NS and various concentrations of VC for 30 min.

As a reducing agent, VC can interact with MnO_2_ NS to produce manganese ions (Mn^2+^) [31]. Based on this property, we hypothesized that VC could reverse the fluorescence quenching of Hb−Fe NCs caused by MnO_2_ NS. Figure 8A illustrates the fluorescence emission spectra of Hb−Fe NCs incubated with 0.05 mg/mL of MnO_2_ NS alongside varying concentrations of VC. It is evident that as the concentration of VC increased from 0 to 1.0 mM, the fluorescence quenching induced by MnO_2_ NS gradually diminished. Figure 8B presents the corresponding UV−Vis absorption spectra of Hb−Fe NCs incubated with MnO_2_ NS and different concentrations of VC. The absorption band associated with MnO_2_ NS significantly decreased as VC concentrations rose from 0 to 1.0 mM, indicating the degradation of MnO_2_ NS. Additionally, the color of the Hb−Fe NCs−MnO_2_ NS solution lightened after incubation with 0.5 mM VC under visible light, and the quenched red fluorescence of the Hb−Fe NCs−MnO_2_ NS solution partially recovered following incubation with 0.5 mM VC under UV light (Figure 6B).

**Figure 9:**
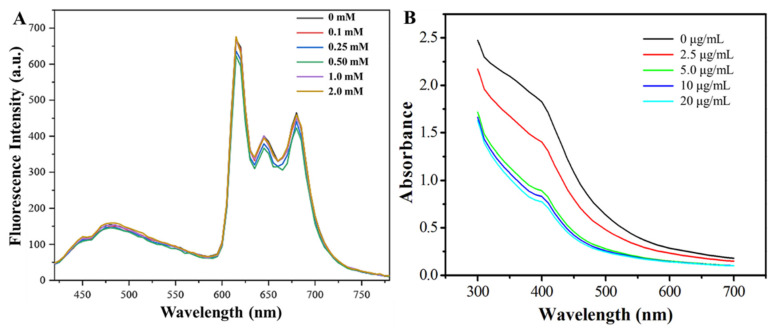
(**A**) The fluorescence emission spectra of Hb−Fe NCs incubated with 0.050 mg/mL of MnO_2_ NS and varying concentrations of AP for 30 min. (**B**) The UV−Vis absorption spectra of Hb−Fe NCs incubated with 0.050 mg/mL of MnO_2_ NS, 0.5 mM of AP, and varying concentrations of ALP for 60 min.

Conversely, AP, which is capable of releasing VC through the hydrolysis of ALP, exhibited a negligible impact on the quenched fluorescence of the Hb−Fe NCs and MnO_2_ NS system within the AP concentration range of 0 to 2.0 mM (see Figure 9A). The notable distinction between VC and AP can be leveraged to develop a detection system for the assessment of ALP, integrating Hb−Fe NCs and MnO_2_ NS.

### 3.2. The Detection of ALP Based on Hb−Fe NCs−MnO_2_ NS-AP System

**Figure 10:**
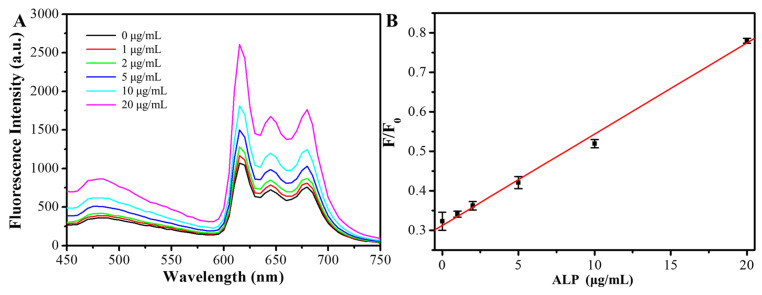
(**A**) The fluorescence emission spectra of Hb−Fe NCs incubated with 0.050 mg/mL of MnO_2_ NS, 0.5 mM of AP, and varying concentrations of ALP for 60 min. (**B**) The data plot of the fluorescence intensity ratio F/F_0_ and ALP concentrations.

As depicted in Scheme 1, we have proposed a system comprising Hb−Fe NCs and MnO_2_ NS for the fluorescent detection of ALP. We examined the fluorescence variations of Hb−Fe NCs when incubated with 0.050 mg/mL of MnO_2_ NS, 0.5 mM of AP, and varying concentrations of ALP over a duration of 60 min under excitation at 400 nm. The results presented in Figure 10A indicate that the fluorescence quenching of Hb−Fe NCs by MnO_2_ NS progressively recovered as the concentration of ALP increased from 0 to 20 μg/mL. Furthermore, we analyzed the correlation between the fluorescence emission intensity at 615 nm and the concentrations of ALP added. Figure 10B illustrates that the relationship between the ratio of fluorescence intensity (F/F_0_) and ALP concentrations can be accurately described by a linear equation.

F/F_0_ = 0.31272 + 0.02312[ALP], μg/mL (3)

**Figure 11:**
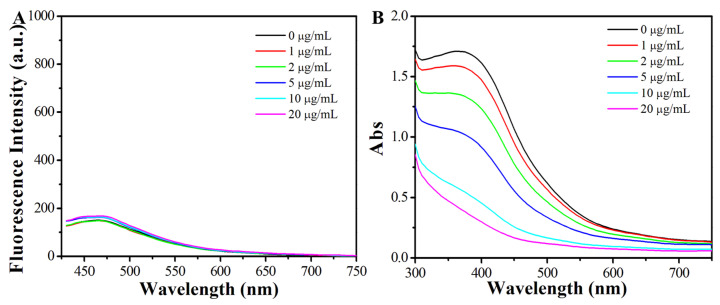
(**A**) The fluorescence emission spectra of mixture composed of MnO_2_ NS (0.050 mg/mL) and AP (0.5 mM) incubated with different concentrations of ALP for a duration of 60 min. (**B**) UV−Vis absorption spectra of mixture composed of MnO_2_ NS (0.050 mg/mL) and AP (0.5 mM) incubated with different concentrations of ALP for a duration of 60 min.

The fit coefficient obtained in this study is 0.999, and the detection limit for ALP is established at 0.6 μg/mL, as determined by the 3σ/k criteria. F_0_ represents the fluorescence intensity at 615 nm of the original Hb−Fe NC solution, which does not contain MnO_2_ NS, AP, or ALP. Conversely, F denotes the fluorescence intensity at 615 nm of the Hb−Fe NC solution that has been incubated with 0.050 mg/mL of MnO_2_ NS, 0.5 mM of AP, and varying concentrations of ALP for a duration of 60 min. As illustrated in Scheme 1, it is posited that the observed fluorescent recovery of Hb−Fe NCs following the addition of ALP is attributable to the production of VC and the degradation of MnO_2_ NS. Additionally, the UV–Vis absorption spectra of the Hb−Fe NC solution, which was incubated with MnO_2_ NS (0.050 mg/mL), AP (0.5 mM), and different concentrations of ALP, were recorded. Figure 9B indicates a significant reduction in the absorbance band around 380 nm, which is associated with MnO_2_ NS, as the concentration of ALP increased. This decrease in absorbance further corroborates the degradation of MnO_2_ NS. To further validate the degradation of MnO_2_ NS induced by ALP, the effect of varying ALP concentrations on the mixture of MnO_2_ NS (0.050 mg/mL) and AP (0.5 mM) was examined. As depicted in Figure 11A, the solution containing MnO_2_ NS and AP exhibited only a minimal fluorescence emission peak around 460 nm, with the addition of ALP exerting negligible influence on the fluorescence. In contrast, the absorption peak at 380 nm progressively diminished as the ALP concentrations increased from 0 to 20 μg/mL (Figure 11B). These findings further substantiate that the fluorescence quenching caused by MnO_2_ NS is a result of IFE, and that the degradation of MnO_2_ NS induced by ALP can restore the quenched fluorescence.

**Figure 12:**
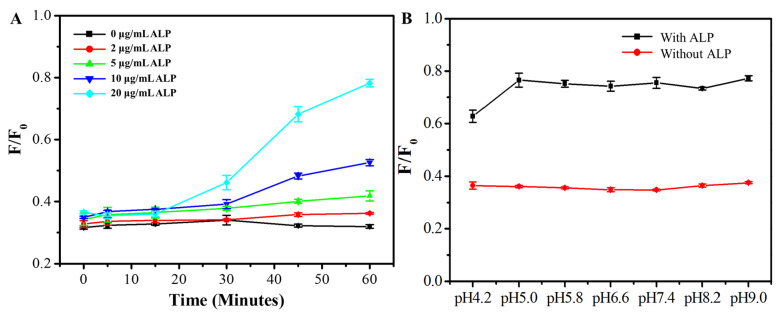
(**A**) The fluorescence intensity at 615 nm of Hb−Fe NCs incubated with 0.050 mg/mL of MnO_2_ NS, 0.5 mM of AP, and varying concentrations of ALP at different times. (**B**) The fluorescence intensity at 615 nm of Hb−Fe NCs incubated with 0.050 mg/mL of MnO_2_ NS, 0.5 mM of AP, and 20 μg/mL of ALP for 60 min in different pH environments.

Figure 12A illustrates the fluorescent variations of the Hb−Fe NCs−MnO_2_ NS−AP system when incubated with varying concentrations of ALP at 0, 2, 5, 10, or 20 μg/mL over a 60-min period. The data indicate that the fluorescence intensity ratio (F/F_0_) of the Hb−Fe NCs−MnO_2_ NS−AP system remained constant in the absence of ALP throughout the 60 min. Conversely, the F/F_0_ ratio of the system increased progressively with the addition of ALP, correlating with the extended incubation time. The fluorescent recovery of the Hb−Fe NCs−MnO_2_ NS−AP system was found to be dependent on both the concentration of ALP and the duration of incubation. Additionally, we examined the influence of pH on the fluorescent recovery of the Hb−Fe NCs−MnO_2_ NS−AP system. Figure 12B presents the fluorescence intensity ratio (F/F_0_) of the Hb−Fe NCs−MnO_2_ NS−AP system, both in the absence and presence of 20 μg/mL ALP, across a range of pH levels from 4.2 to 9.0. The results demonstrate that ALP exhibited limited recovery capability in an acidic environment (pH 4.2), while the fluorescence recovery effect of the Hb−Fe NCs−MnO_2_ NS−AP system, induced by ALP, progressively improved as the pH increased from 5.0 to 9.0.

**Figure 13:**
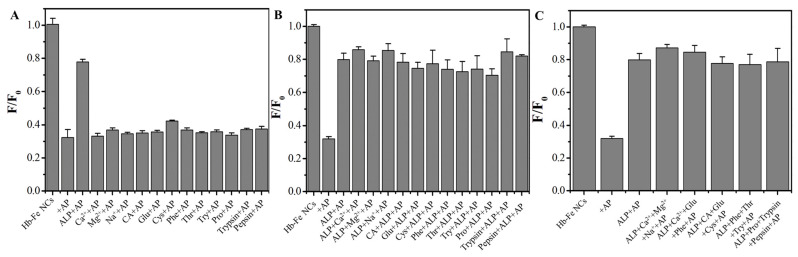
(**A**) The fluorescence intensity ratio, denoted as F/F_0_, of the Hb−Fe NCs−MnO_2_ NS−AP system incubated with various kinds of metal ions or biomolecules for 60 min. (**B**) The fluorescence intensity ratio F/F_0_ of the Hb−Fe NCs−MnO_2_ NS−AP system incubated with 20 μg/mL ALP alongside other metal ions and biomolecules for 60 min. (**C**) The fluorescence intensity ratio F/F_0_ of Hb−Fe NCs−MnO_2_ NS−AP system incubated with 20 μg/mL ALP and mixed solution composed of various metal ions and biomolecules. Hb−Fe NCs at 1.65 mg/mL, MnO_2_ NS at 0.05 mg/mL, AP at 0.5 mM, 20 mM Tris-HCl buffer pH 7.4.

In addition, we examined the selectivity of the Hb−Fe NCs−MnO_2_ NS−AP system for the detection of ALP. As illustrated in Figure 13A, the Hb−Fe NCs−MnO_2_ NS−AP system was incubated with various substances, including ALP (20 μg/mL), Ca^2+^ ions (0.2 mM), Mg^2+^ ions (0.2 mM), Na^+^ ions (0.2 mM), citric acid (CA) (0.2 mM), glucose (Glu) (0.2 mM), cysteine (Cys) (0.2 mM), phenylalanine (Phe) (0.2 mM), threonine (Thr) (0.2 mM), tyrosine (Tyr) (0.2 mM), proline (Pro) (0.2 mM), trypsin (50 μg/mL), and pepsin (50 μg/mL), each tested individually. The findings indicated that only ALP significantly induced the fluorescent recovery of the Hb−Fe NCs−MnO_2_ NS−AP system, while the other metal ions and biomolecules exhibited minimal impact. The selectivity for ALP is attributed to its catalytic function in the hydrolysis of AP substrates. Additionally, we assessed the detection performance of the Hb−Fe NCs−MnO_2_ NS−AP system for ALP in the presence of other metal ions and biomolecules. As depicted in Figure 13B, the presence of Ca^2+^ ions, Mg^2+^ ions, Na^+^ ions, CA, Glu, Cys, Phe, Thr, Tyr, Pro, trypsin, or pepsin did not influence the fluorescent response of the Hb−Fe NCs−MnO_2_ NS−AP system to ALP. Furthermore, we evaluated the detection performance of the Hb−Fe NCs−MnO_2_ NS−AP system for ALP in mixed solutions containing various metal ions and biomolecules, including mixed solution I (Ca^2+^ ions, Mg^2+^ ions, and Na^+^ ions), mixed solution II (Ca^2+^ ions, Glu, and Phe), mixed solution III (CA, Glu, and Cys), mixed solution IV (Phe, Thr, and trypsin), and mixed solution V (Pro, trypsin, and pepsin). Figure 13C demonstrated that the Hb−Fe NCs−MnO_2_ NS−AP system maintained its responsiveness to ALP even in the complex mixtures containing metal ions and biomolecules. The above results demonstrated the selectivity and anti-interference capabilities of the Hb−Fe NCs−MnO_2_ NS−AP system for ALP detection.

### 3.3. The Application in Real Samples

To assess the practicality of this method for the detection of ALP in actual samples, we measured ALP levels in fetal bovine serum utilizing the Hb−Fe NCs−MnO_2_ NS−AP system. Various concentrations of ALP were introduced into a fivefold-diluted fetal bovine serum solution, and the resultant ALP levels were subsequently quantified using the aforementioned system. As indicated in Table 2, the recovery rates for ALP ranged from 98% to 103%, with relative standard deviations (RSDs) not exceeding 3.6%. These findings demonstrated the potential applicability of this method for ALP detection in real samples.

## 4. Conclusions

We synthesized red-emitting Hb−Fe NCs and investigated the IFE occurring between the Hb−Fe NCs and the MnO_2_ NS system. The pronounced fluorescence quenching of the Hb−Fe NCs, induced by the MnO_2_ NS, can be reversed through the addition of VC and the subsequent degradation of the MnO_2_ NS. Based on the hydrolysis of AP catalyzed by ALP, we further developed the Hb−Fe NCs−MnO_2_ NS−AP system, which was effectively employed for the fluorescent detection of ALP within a concentration range of 0–20 μg/mL. Given the fluorescence recovery observed in the Hb−Fe NCs−MnO_2_ NS−AP system, we anticipate that this proposed system may be applicable for monitoring fluctuations in ALP levels in more complex biological environments, such as within cells and organs.

## Figures and Tables

**Table 1:** The relative parameters of the fitted curve of Hb−Fe NC solution, Hb−Fe NCs−MnO_2_ NS solution.

	A_1_	τ_1_	A_2_	τ_2_	R^2^
Hb−Fe NCs	553.6	5.529	279.6	0.8325	0.9316
Hb−Fe NCs−MnO_2_ NS	463.3	6.490	385.1	1.350	0.9409

**Table 2:** The detection of ALP in fetal bovine serum samples.

Samples	Added (μg/mL)	Found (μg/mL)	Recovery (%)	RSD (*n* = 3, %)
1	5.0	4.9	98%	1.2%
2	10.0	10.3	103%	3.6%

## Data Availability

Data are contained within the article.
